# Airway Smooth Muscle as a Target in Asthma and the Beneficial Effects of Bronchial Thermoplasty

**DOI:** 10.1155/2012/593784

**Published:** 2012-09-16

**Authors:** Luke J. Janssen

**Affiliations:** Firestone Institute for Respiratory Health, St. Joseph's Hospital and Department of Medicine, McMaster University, Hamilton, ON, Canada L8N 3Z5

## Abstract

Airflow within the airways is determined directly by the lumenal area of that airway. In this paper, we consider several factors which can reduce airway lumenal area, including thickening and/or active constriction of the airway smooth muscle (ASM). The latter cell type can also contribute in part to inflammation, another feature of asthma, through its ability to take on a synthetic/secretory phenotype. The ASM therefore becomes a strategically important target in the treatment of asthma, given these key contributions to the pathophysiology of that disease. Pharmacological approaches have been developed to elicit relaxation of the ASM, but these are not always effective in all patients, nor do they address the long-term structural changes which impinge on the airway lumen. The recent discovery that thermal energy can be used to ablate smooth muscle has led to the development of a novel physical intervention—bronchial thermoplasty—in the treatment of asthma. Here, we review the evolution of this novel approach, consider some of the possible mechanisms that account for its salutary effects, and pose new questions which may lead to even better therapies for asthma.

## 1. The Airway Lumen: Physiological Importance

 The primary function of the lungs is to meet the metabolic demands of the body by absorbing atmospheric oxygen, delivering that to the rest of the body, and excreting carbon dioxide. The importance of this function is reflected in the amount of oxygen required at rest and during exercise. An average 70 kg individual has a resting oxygen uptake of 250 mL/min, which for a number of reasons requires a ventilation rate of 7-8 L/min. First and foremost, alveolar ventilation is very inefficient due to the ventilatory anatomic dead space: that is, the average individual has a resting tidal volume of 300–400 mL (and rate of 15–20 breaths per minute), but gas exchange occurs almost exclusively in the alveoli, with minimal uptake in the conducting airways. Also, it needs to be kept in mind that approximately four fifths of that inspired volume is not useful (atmospheric air is only 20.93% oxygen) and that breathing itself requires effort. If that individual walks briskly at 4-5 km/hour, oxygen uptake goes to 1000 mL/min, requiring about 30 L/min of ventilation. More strenuous exertion (e.g., running up a flight of stairs) can demand ventilatory rates of 125–150 L/min (the metabolic costs are the same for normals and athletes, but the latter are able to reach much higher ventilation rates and power outputs).

 Poiseuille's Law relates together the various factors which determine the flow *F* of a fluid of a given viscosity (*η*) through a tube (radius *r*; length *L*) driven by a pressure gradient Δ*P*), as follows:

(1) F=(ΔPπr4)(8ηL).

A very compelling message to be obtained from this equation is the dramatic effect that narrowing of the vessel lumen has on fluid flow: a change in vessel radius of only 10% can result in a decrease in flow of 36% (1–0.9^4^), while a narrowing of 20% leads to a 59% reduction in flow (1–0.8^4^) (this mathematical consideration does not take into account turbulence at the vocal cords and upper airways; elastic and inertial adjustments related to the mass of the chest and abdomen that must also move during breathing, etc.). There are many factors which can directly influence airway lumen diameter, as outlined in the following.

## 2. Airway Wall Thickening 

 Mathematical modelling has shown how changes in airway wall geometry alone—more precisely, thickening of the airway wall—can seriously hinder airflow [1, 2]. The airway wall consists of an epithelial layer founded upon a basement membrane, with a band of airway smooth muscle (ASM) encircling both: changes in all three of these components are known to contribute to wall thickening and, ultimately, to asthma.

### 2.1. Basement Membrane

The basement membrane and extracellular matrix are made up of various proteoglycans, glycosaminoglycans, and connective tissue proteins (collagen, elastin, fibronectin, etc.). This layer provides structural integrity and a platform on which other cells (epithelium; ASM; inflammatory cells) can reside and/or migrate. More importantly, there is an abundance of studies which have shown increased amounts of connective tissue proteins in the airway wall and underneath the basement membrane layer in asthma; these will not be detailed here but have been reviewed elsewhere [3]. 

### 2.2. Epithelial (Goblet) Cell Hyperplasia and Increased Mucous Secretion

**Figure 1:**
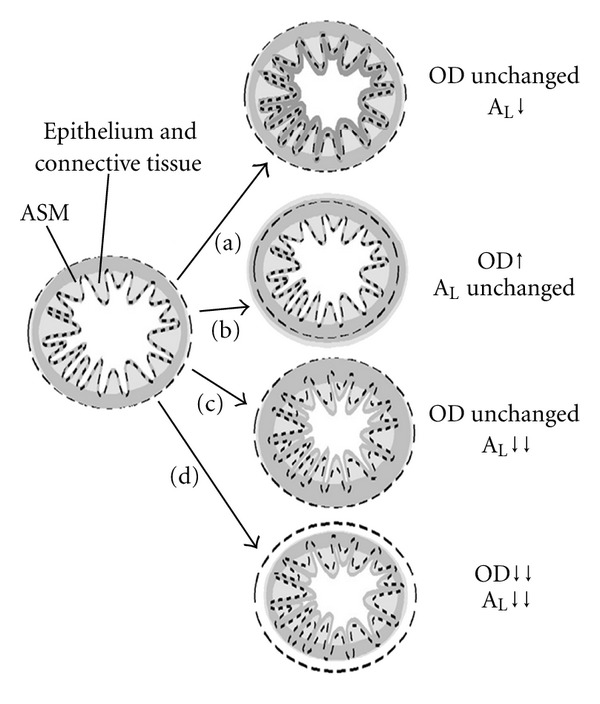
Image on the left depicts a hypothetical airway: dashed lines on the left and right images circumscribe the outer and inner dimensions prior to various changes which can impact airway lumenal area (A_L_) and airflow. (a) Thickening of the basal lamina, epithelial cell hyperplasia, edema formation, and/or bronchial vascular dilation all lead to a swelling or thickening of the innermost layer(s) of the airway: outer diameter is unchanged, but A_L_ is reduced, leading to decreased airflow. On the other hand, the ASM layer itself might become thickened: this can be directed outwardly such that A_L_ and airflow are unchanged (b) or directed inwardly such that A_L_ and airflow are both reduced (c). Finally, the ASM can actively constrict, leading to a reduction in outer diameter, A_L_ and airflow (d).

 An epithelial layer lines the lumenal face of the airways and comprises up to 8 morphologically distinct cell types, the anatomy and functions of which have been described in greater detail elsewhere [4]. However, one of these cell types which is particularly relevant to the current discussion is referred to as the mucous cell or goblet cell. In asthmatics and in many animal models of airway hyperresponsiveness (routinely used to better understand the changes seen in asthma), there is thickening of the epithelial layer due to goblet cell hyperplasia [5, 6]. The latter protrude into the lumen and also secrete a thick mucous: both of these changes lead to an effective decrease in lumenal area (Figure 1(a)) and, thus, to decreased air flow. In some cases, particularly fatal asthma, mucous plugs can completely obliterate airflow.

### 2.3. Increased ASM Mass

 In asthma and in many animal models of airway hyperresponsiveness, the ASM cell layer which encircles the airway wall can also be greatly thickened [7, 8]. There has long been discussion as to whether this is due to hypertrophy (increased cell size) *versus* hyperplasia (increased cell number), with evidence available on both sides of the argument in animal and human preparations [9–11]. However, a recent study which focussed specifically on this question in human airway tissues obtained from normal control volunteers as well as nonfatal and fatal asthmatics concluded that hypertrophy accounts for the thickening in the large airways of both asthmatic groups, while hyperplasia only occurred in fatal asthma [12].

 While stereological morphometric and statistical analytical techniques are available to determine whether ASM thickening is due to hyperplasia *versus* hypertrophy, it is not yet possible to determine whether the thickening is primarily directed outwardly (i.e., away from the lumen) and/or inwardly (i.e., towards the lumen) over the months and years as asthma develops. These two changes have very different functional consequences. That is, the resting lumenal area is unchanged in the former case (Figure 1(b)) but markedly decreased in the latter case (Figure 1(c)). In contrast, both changes are associated with increased contractile responses which can also obstruct air flow (next section).

## 3. Active Constriction of the ASM 

 For many decades, the primary function of ASM was seen to be the same as that of all other muscles: to constrict and thereby generate tension and/or shortening. However, over the past decade, questions have been raised as to what would be the physiological purpose of this mechanical response in the airways, and what then is the role of ASM in normal lung physiology [13, 14]; other possible physiological roles for ASM will be mentioned briefly (Section 4) and have been reviewed in more detail elsewhere. Nonetheless, the ASM is able to constrict in response to a wide variety of physiological and pathological stimuli and thereby effect a profound decrease in airway lumen diameter (Figure 1(d)). The increased ASM mass seen in asthmatics and in animal models of airway hyperresponsiveness (AHR) is expected, then, to lead to increased bronchoconstriction. Increased sensitivity to a variety of inhaled spasmogens (methacholine, histamine, etc.) is well documented in asthmatic individuals [9, 10, 15–22]. However, there is far less consensus as to whether the ASM cell *per se* functions differently in asthma. While many studies using isolated tissues/cells from allergen-induced *animal models* have found larger responses at any given spasmogen concentration (hyperreactivity) and/or a distinct leftward shift in the concentration-response curves for various spasmogens (hypersensitivity) [23–37], studies using *human* airway tissues and cells are far less clear. One study of tracheal smooth muscle obtained from severe asthmatics found increased sensitivity to cholinergic stimulation or to histamine and impaired relaxations to isoproterenol [38], while two other studies using small bronchi from mild [15] or severe [39] asthmatics found no changes in sensitivity to excitatory stimuli (although the potency of isoproterenol was still decreased ~10-fold [39], possibly due to desensitization following frequent use of inhaled *β*-agonist). Single cells obtained by bronchial biopsy from asthmatic patients showed increased shortening capacity, shortening velocity, and expression of myosin light chain kinase (MLCK) [40]. Bronchial cells cultured from asthmatics show decreased SERCA2 expression and altered Ca^2+^-handling [41] and retain a hyperproliferative and hypersecretory phenotype through repeated rounds of cell passaging [42]. Thus, there are reasons to question whether basic contractile signalling mechanisms in ASM are altered in asthma.

## 4. Nonmechanical Functions of ASM

 ASM cells are now known to subserve a number of other functional activities, including synthesis and secretion of extracellular matrix proteins and proinflammatory mediators and antigen presentation [43–47]. As such, the ASM itself can contribute in several ways to the wall remodelling and inflammation which characterize asthma; ASM cells from patients with asthma display greater proliferative and synthetic responses compared to ASM cells from nonasthmatic subjects [48]. *In vitro *studies using cultured cells have shown that ASM cells which take on or manifest the secretory phenotype show reduced expression of the contractile proteins *α* smooth muscle actin and/or smooth muscle myosin. This may explain, in part, why there can be increased ASM mass in asthma but little (or none?) change in the contractile responses of excised tissues. It is unknown whether ASM cells which are able to present antigen also lose the contractile phenotype.

## 5. The ASM Is a Key Target in Asthma 

 The symptoms and morbidity associated with asthma are in part a result of episodic bronchoconstriction, which is believed to be largely due to an underlying AHR. One way to quantify pulmonary function is to have the individual inhale fully (“total lung capacity”), then to exhale forcibly and maximally, and measure the volume of air which is expelled within the first second (“forced expiratory volume,” or FEV_1_). One measure of airway responsiveness to an agonist is to have that individual first inhale an aerosol containing a bronchoconstrictor agent (e.g., methacholine, histamine, etc.) and determine the concentration of agonist which decreases FEV_1_ by 20% (PC_20_). Improvement in asthma severity (e.g., as a result of allergen avoidance [49, 50] or treatment with inhaled corticosteroids [51–53]) is often associated with an improvement of PC_20_ by 1-2 doubling concentrations. These induced changes are small in comparison to the difference in PC_20_ between asthmatic and nonasthmatic populations. Although the magnitude of AHR can fluctuate within an individual, there appears to be limits to the extent to which it can be improved even with optimal disease management based on current guidelines. Moreover, in patients optimally managed under research study conditions, there are reported symptoms on almost 50% of study days, and over 15% of these individuals still experience at least one severe exacerbation per year [54]. Altogether, it is clear that the mechanisms underlying AHR and airway dysfunction are complex and that not all of these are addressed by current treatment practices.

 In an attempt to address the aspects of asthma resistant to current therapies, new pharmacological treatments have been developed based largely on our improved understanding of disease mechanisms [55, 56]. Much of the acute and life-threatening changes are due to ASM contraction causing airflow obstruction [38, 57]. Asthmatics carry symptom-relieving inhaled medications, such as a rapidly acting bronchodilator [55, 56, 58], which act within minutes—a timeframe more likely related to muscle relaxation and bronchodilation than to reversal of edema, elimination of mucus, reduction of wall thickening, or relief of any other mechanism by which inflammation causes airway obstruction. These medications are thus extremely valuable for treating asthma but do not modify the disease state: once these medications are stopped, their benefit rapidly wanes. Asthma is also characterized by airway inflammation, to which the ASM also plays a contributory role in that it can synthesize and secrete proinflammatory mediators and present antigen. Altogether, then, the ASM plays a key role in the pathophysiology underlying asthma (while seemingly playing no important or useful role in normal physiology [13, 14]) and is therefore a prime target in our search for better strategies to treat asthma: any approach which selectively decreases ASM mass (and its consequent ability to constrict the airway and contribute to inflammation) could be superior to the current strategy of treating its symptoms. This tantalizing prospect led to ideas of interfering with ASM proliferation or migration, or promoting ASM apoptosis or delivering toxins to the ASM *per se* using immunological or genetic approaches [59]. Thermal energy has been used to reduce smooth muscle mass in other disease states, which led some to try this approach in asthma. In the next two sections, we will summarize that foundational work in non-ASM preparations (Sections 6 and 7), as well as more recent and encouraging data showing its usefulness in ASM and asthma. 

## 6. Response of Smooth Muscle to Thermal Injury 

 Many have examined the response of smooth muscle to relatively mild heat stress (HS) (<43°C), too numerous to cite here: these describe typical febrile responses including induction of the heat shock protein cascade. However, this level of thermal exposure is not known to be associated with loss of smooth muscle—airway or otherwise. 

 On the other hand, numerous studies describe the delivery of supraphysiological temperatures (45-65°C) for the treatment of benign prostatic hyperplasia [60–63]. Histological examination of the prostatic tissues weeks after such thermotherapy revealed loss of smooth muscle mass and dark staining of nuclear chromatin, and isolated segments of these tissues showed decreased responsiveness [63]; it is important to note that the degree of these structural and functional changes was not seen at temperatures below 48°C and was thermal “dose” dependent [63]. In another study of the response to HS in the guinea-pig vas deferens, there was loss of adrenoceptors, loss of myofilaments, and dark staining of nuclear chromatin of the smooth muscle cells, again only at temperatures in excess of 50°C [62]. 

 The therapeutic application of HS to coronary arteries has been attempted in the past: “thermal balloon angioplasty” involved introduction of a balloon catheter which was then inflated and heated to temperatures ranging from 50 to 100°C [64–69]. This resulted in reduced vasoreactivity but also produced intimal hyperplasia and fibroproliferation leading to restenosis: this therapy was soon abandoned when it became apparent that the latter long-term structural changes outweighed the short-term functional benefits. 

## 7. Bronchial Thermoplasty 

 Recently, the direct application of thermal energy was found to be useful in reducing bronchial wall muscle content in asthma [70–75]. While under only local anaesthesia, a four-armed basket electrode is introduced into the airways using a bronchoscopic catheter, expanded to make contact with the airway wall and then used to deliver radiofrequency energy in order to warm the airway wall to a target temperature of 65°C (coffee or tea can be imbibed at higher temperatures than this). The outcome of this procedure—referred to as bronchial thermoplasty (BT)—is an airway which looks normal with respect to epithelium and basement membrane (no evidence of scar tissue) but which now displays approximately 50% less ASM (at least 3–6 weeks following BT) [74] (although it should be pointed out that this study was done in nonasthmatics who were scheduled to undergo lung resection for carcinoma, and it could be argued that inflamed asthmatic ASM may respond differently to HS). Loss of ASM should be associated with reduced potential for bronchoconstriction: indeed, patients with mild, moderate, and severe asthma have been successfully treated, demonstrating persistent improvement in asthma control, better quality of life scores and fewer exacerbations which persist for several years [76–81]. One of the first clinical trials, done in 32 severe asthmatics, found no significant improvement in PC_20_ after 1 year [79], although another larger, 5-year trial with 101 moderate-to-severe asthmatics noted improvement in PC_20_ values in years 2 and 3 [81]; a third 1-year clinical study with 288 severe asthmatics made no comment about PC_20_ values [76]. Also, it has not yet been demonstrated in human lungs that the ASM does not return following BT. The adverse events associated with BT were encountered in the peritreatment period—there were no long-term adverse outcomes such as progressive tissue changes or airway injury. This experience shows that human airways can tolerate thermal interventions, that long-term complications are rare, that benefits can persist for years, and that reduction of smooth muscle content of the airways in humans is safe and feasible. 

 All of these points notwithstanding, there are limitations associated with BT. First and foremost, there are uncertainties regarding the long-term outcome(s) of this procedure, other than reduction of ASM mass [74]: the cellular/molecular changes which it induces within the lung have yet to be explored in detail. Second, BT is a time-consuming procedure: the bronchial catheter must be inserted into every accessible airway and withdrawn stepwise (in order to allow the basket to make contact with the entire length of each airway) for 10 seconds at each step. There is obviously a considerable degree of invasiveness inherent in this approach, and therefore certain risks are inevitable. Also, this procedure can only be performed on airways which are larger than the diameter of the bronchial catheter, which is unfortunate since smaller airways may also play a role in clinically relevant resistance to airflow. 

## 8. How Does BT Work?

 The response to HS is a phylogenetically ancient cellular response to exogenous stress (including high temperatures) which leads to binding of HS-activated transcription factors to cis-acting HS-response elements found within the promotor regions of a number of genes, including HSPs [82] and cytokines [83]. HSPs have been found in every species in which they have been sought [82]. In general, they function as molecular chaperones, binding to proteins which are not in their native conformation due to denaturing stresses (such as heat) or which are not yet fully modified following *de novo* synthesis and thus restore function. They can also contribute to regulation of the intracellular matrix, as they have recently been shown to do in ASM stimulated with physiological autacoids [84, 85]. Generally speaking, HSPs are taken to be beneficial and/or protective in nature. However, others have shown they can also exacerbate injurious events: for example, while febrile-range hyperthermia accelerated pathogen clearance and increased survival in experimental *Klebsiella pneumoniae* peritonitis, it also accelerated lethal lung injury in a mouse model of pulmonary oxygen toxicity, leading the authors to suggest that the lung may be particularly susceptible to the injurious effects of hyperthermia [86, 87]. Likewise, there can be a bimodal effect of HSPs, with decreased protection or even deleterious effects when HSPs are overexpressed [82] (as would occur following such a severe thermal injury). Febrile-range hyperthermia (~42°C, 120 min) of cultured airway epithelial cells triggers an HSP response followed by increased expression of interleukin-8/CXCL-8 [88, 89]. 

 On the other hand, it may be that HS upsets the balance of removal and renewal of the ASM, by triggering some kind of apoptotic response, or disrupting ASM proliferation and/or migration. We have examined the cellular structural responses to HS caused by brief (30–60 seconds) immersion of excised ASM tissues into a physiological buffer medium warmed to various temperatures as a surrogate for BT. Using TUNEL and immunohistochemical analytic techniques (for DNA laddering and caspase 3 activation), we found significant cell death in all tissues heated to 65°C and limited cell death at lower temperatures. Similarly, NADH diaphorase activity (another measure of cell death) was reduced at 55°C and abolished at higher temperatures. 

 Prior to that study, we had used the thermal immersion model of BT to study the immediate effects on ASM mechanical function [90]. We found that contractile responses to millimolar potassium chloride or acetylcholine could be completely abolished within seconds of treatment using 55°C or higher, whereas temperatures of 50°C or lower were relatively inconsequential (unless much longer treatment durations were employed). The rapid onset of this response to heat treatment (timeframe of seconds) ruled out the interpretation that the apoptotic cascade or heat shock proteins are involved in mediating the loss of contractility, since both processes require expression of various proteins (timeframe of many hours). Instead, our data supported the interpretation that the immediate loss of functionality was due to denaturation of the myosin molecules, possibly preventing ATP hydrolysis and/or their interaction with actin.

 Other temperature-sensitive mechanisms may be involved in the response to BT. One subclass of transient receptor potential (TRP) channels is activated by warmth (25–40°C; TRPV3 and TRPV4), febrile temperatures (>43°C; TRPV1 [91]), or noxious heat (>52°C; TRPV2 [92]). Involvement of one or more of these would be evidenced by a very sharp thermal sensitivity profile and short latency (on the order of milliseconds). TRPV1 and TRPV2 channels have indeed been identified in ASM [93], although there is no clear *a prior* reason to expect that their activation would lead to loss of mechanical function. 

 On the other hand, a much earlier study found MLCK to be irreversibly inactivated by Ca^2+^/calmodulin-dependent kinase II (CamK-II) in a steeply temperature-dependent manner, with half-maximal effect at ~55°C when [Ca^2+^] in the enzyme reaction assay was high (lower temperatures were effective at lower [Ca^2+^]) [94]. Inactivation of MLCK could easily account for loss of mechanical function.

 BT may also be reducing the amount of vascular smooth muscle mass in the airway wall: there is some evidence that vascularization of the airway wall is increased in some forms of asthma [95, 96], and others have suggested that dilation of that vascular bed contributes in part to asthma induced by exercise or inhalation of cool, dry air.

 Finally, it may be that BT leads to a functional denervation. The earliest histological data obtained during the development of BT as a clinical tool reported that the airway wall a few weeks following thermal injury appeared normal apart from a notable reduction in ASM fibers: the epithelium appeared to be unaffected (although this may have been repopulated by new/other cells). However, neither histological nor functional studies were performed to determine whether nerve fibers and varicosities were also normal. This too is relevant in that (i) the ASM receives strong excitatory neural input from the cholinergic innervation, and cholinergic receptor antagonists (atropine; ipratropium; tiotropium) have proven to be useful in asthma; (ii) sensory neuronal axon reflex can lead to powerful bronchoconstriction [97].

## 9. Conclusion and New Directions

 Altogether, then, studies using animal tissues which had been immersed in heated medium to induce HS suggest that BT in humans may be associated with an immediate (within seconds) loss of ability on the part of the ASM to generate a mechanical response, induction of cell death over the next 1–24 hours, and a marked reduction in ASM mass over the ensuing weeks and months. The net result is expected to be widening of the airways and lessened ability of the airways to actively constrict (although this has not been conclusively demonstrated in humans), thereby removing those geometrical burdens on airflow and possibly also a lessening of the airway inflammation (since the ASM can be a source of inflammatory mediators) which accompanies and exacerbates asthma. More importantly, there is an improvement in quality of life scores, and fewer exacerbations.

 The precise mechanism(s) by which BT produces its beneficial effects are far from clear and require extensive further studies. In particular, the exact pathway(s) that lead to loss of airway smooth muscle—various apoptotic responses, autophagy, necrotic cell death, and so forth—are far from clear. A better understanding of these questions may lead to enhancements in the delivery of the thermal injury or, even better, substitution of this physical approach with some form of chemical/pharmacological agent which will trigger those pathways directly. The latter may be better in that they might be easily inhaled, and it would then be possible to ablate ASM in all the airways (large and small; upper lobes as well as lower lobes), rather than just a large fraction of the ones which are easy to reach with a bronchoscope. 

 Numerous other interesting and imperative questions remain. Why does the ASM layer not return following BT? Does BT somehow alter the airway wall, particularly the connective tissue matrix, such that new ASM cells do not migrate in? Does it somehow alter the remaining ASM cells which survive BT such that they do not proliferate? If so, how? What effects does BT have on the innervation? On fibroblasts? 

 Answers to these questions may eventually lead to an actual “cure” for asthma, rather than the current approaches which seek to treat its symptoms (bronchodilators and anti-inflammatories).

## Abbreviations

A_*L*_: Airway lumenal area;
AHR: Airway hyperresponsiveness;
ASM: Airway smooth muscle;
BT: Bronchial thermoplasty;
CamK-II: Ca^2+^Calmodulin-dependent kinase II;
Δ*P*: Pressure gradient;
*F*: Flow;
FEV_1_: Forced expiratory volume;
HS: Heat stress;
HSP: Heat shock proteins;
*L*: Length (of a tube);
MLCK: Myosin light chain kinase;
*η*: Fluid viscosity;
PC_20_: Concentration of agonist which decreases FEV_1_ by 20%;
*r*: Radius;
TRP: Transient receptor potential.
